# Transcutaneous oxygen pressure-guided prophylactic fasciotomy and negative pressure wound therapy for contrast extravasation injury of the hand: A case report

**DOI:** 10.1097/MD.0000000000045413

**Published:** 2025-10-24

**Authors:** Woo Jin Song, Hyun Beom Choi, Ha Jong Nam, Chan Yeong Lee, Hwan Jun Choi

**Affiliations:** a Department of Plastic and Reconstructive Surgery, Soonchunhyang University Seoul Hospital, Soonchunhyang University College of Medicine, Seoul, Korea; b Department of Plastic and Reconstructive Surgery, Soonchunhyang University Gumi Hospital, Gumi, Korea; c Department of Plastic and Reconstructive Surgery, Soonchunhyang University Cheonan Hospital, Cheonan, Korea.

**Keywords:** compartment syndromes, fasciotomy, negative pressure wound therapy, wound healing

## Abstract

**Rationale:**

Contrast medium extravasation injury is a rare but potentially limb-threatening complication of intravenous imaging, particularly in anatomically confined regions such as the dorsum of the hand. Thus, adjunctive diagnostic tools are needed to supplement clinical judgment and identify patients who will benefit from early surgical interventions.

**Patient concerns:**

A 55-year-old female patient presented with extensive swelling and pain in the left hand following contrast medium extravasation during a computed tomography angiography. The initial physical examination revealed minimal skin changes; however, the patient reported progressive pain and taut swelling.

**Diagnosis:**

The examination results were inconclusive of compartment syndrome. Transcutaneous oxygen pressure (TcPO₂) monitoring revealed critically low perfusion values (5 mm Hg in the dorsal wrist and 1 mm Hg in the distal dorsum), suggesting evolving ischemia. Forward-looking infrared thermal imaging confirmed localized hypoperfusion.

**Interventions:**

Based on TcPO₂ and thermal imaging findings, a prophylactic fasciotomy was performed. Negative pressure wound therapy was applied, and primary wound closure was performed on postoperative day 3 without the need for skin grafting.

**Outcomes:**

The patient recovered without wound-related complications, and no additional procedures were required.

**Lessons:**

This may be the first reported case of contrast medium extravasation injury in the hand managed with TcPO₂-guided fasciotomy. TcPO₂ and forward-looking infrared thermography may serve as alternative noninvasive tools to support early decision-making for fasciotomy in cases where traditional clinical signs are absent. Early intervention based on perfusion metrics may prevent irreversible ischemic complications.

## 1. Introduction

Contrast medium extravasation is a relatively common complication of intravenous imaging, occurring in up to 0.9% of cases.^[1]^ While most incidents resolve with conservative care, large-volume extravasation in anatomically confined regions such as the dorsum of the hand can result in severe complications, including tissue necrosis and acute compartment syndrome.^[2]^ Early surgical intervention may be necessary to prevent irreversible damage; however, clinical decision-making is often difficult in the absence of overt signs such as skin discoloration or blistering.^[3]^

To date, no standardized, objective method has been developed to identify patients at risk of evolving ischemia before the development of visible soft tissue compromise.^[4]^ Traditional decision-making relies heavily on subjective clinical examination or, in rare cases, invasive compartment pressure monitoring.^[5]^ Transcutaneous oxygen pressure (TcPO₂), a noninvasive tool widely used in chronic limb ischemia and wound care, provides quantitative real-time information on local tissue perfusion.^[6]^ However, to our knowledge, its application in the setting of contrast extravasation injury has not been previously reported in the medical literature.

Herein, we report what may be the first case of contrast medium extravasation injury in the hand in which TcPO₂ monitoring was used to guide early surgical decompression in the absence of external ischemic signs. The patient was successfully treated using TcPO₂-guided prophylactic fasciotomy, followed by negative pressure wound therapy (NPWT), resulting in full functional recovery without complications. This case highlights the potential of TcPO as an adjunctive decision-making tool for the early identification and management of high-risk extravasation injuries.

## 2. Case presentation

A 55-year-old woman underwent contrast-enhanced computed tomography for routine health screening. During the intravenous injection of 60 mL of nonionic iodinated contrast agent (Iobitridol, Xenetix 350, Guerbet, France) through a peripheral vein on the dorsum of her left hand, the patient immediately reported severe pain, taut swelling, and numbness. The injection was promptly discontinued, and the patient was referred for surgical evaluation the following day.

**Figure 1.**
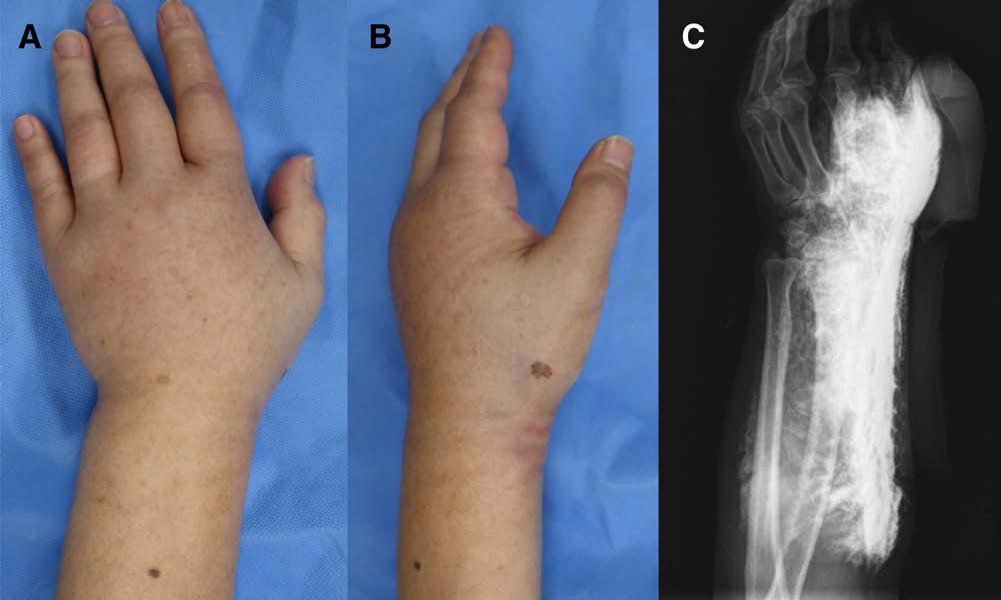
Initial evaluation findings of the reported patient’s left hand ~24 hours after contrast extravasation. (A) Dorsal view showing diffuse swelling with intact skin. (B) Lateral view confirming taut subcutaneous edema without visible color change or bullae. (C) Plain radiograph demonstrating diffuse soft tissue density compatible with extravasated contrast material; no gas formation or bony injury is observed.

On physical examination ~24 hours after the extravasation, the left hand appeared markedly swollen and exhibited tautness of the skin in both the dorsal and lateral aspects, without signs of skin discoloration, blistering, or necrosis (Fig. 1A and B). Capillary refill was borderline delayed, and active finger motion was limited owing to pain. Plain radiography revealed diffuse soft tissue infiltration without subcutaneous air or osseous abnormalities (Fig. 1C).

**Figure 2.**
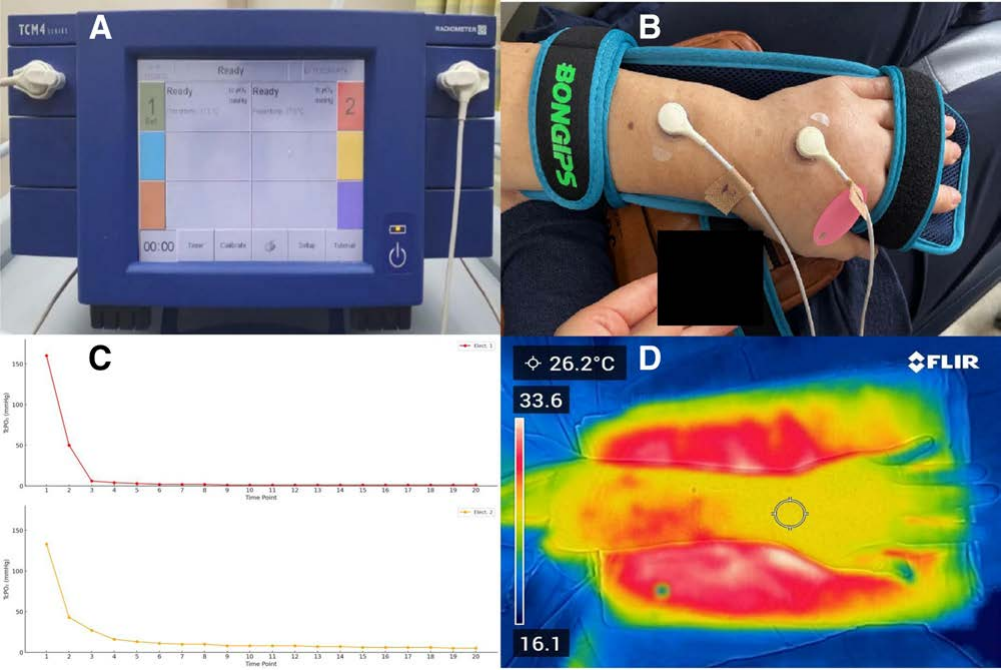
Objective perfusion and temperature assessment prior to fasciotomy. (A) Transcutaneous oxygen pressure (TcPO₂) measuring instrument used in this study (TCM400R, Radiometer Medical ApS, Denmark). (B) TcPO₂ was measured at the distal dorsum and dorsal wrist using skin surface electrodes. (C) TcPO₂ readings demonstrating critically low oxygen tension: 1 mm Hg at the distal dorsum and 5mm Hg at the dorsal wrist. (D) Thermographic image showing a uniform dorsal surface temperature of ~33°C, comparable to surrounding areas, indicating no focal hyperthermia suggestive of inflammation or infection.

To objectively assess tissue viability, we measured TcPO₂ using a surface electrode system with a TCM400R monitor (Radiometer Medical ApS, Denmark), which provides a continuous, noninvasive measurement of transcutaneous oxygen tension (Fig. 2A and B). Critically low values were recorded: 5 mm Hg at the dorsal wrist and 1 mm Hg at the distal dorsum of the hand (Fig. 2C). Thermographic imaging was performed using a handheld infrared (i.e., forward-looking infrared [FLIR]) camera to further evaluate for possible infection or inflammatory response, revealing a surface temperature of ~33°C, consistent with surrounding tissue and within normal physiologic range (Fig. 2D). This finding supported the absence of localized hyperemia or inflammatory reactions. Collectively, the examination findings indicated impending ischemia despite the lack of overt cutaneous signs, and emergency surgical intervention was planned.

**Figure 3.**
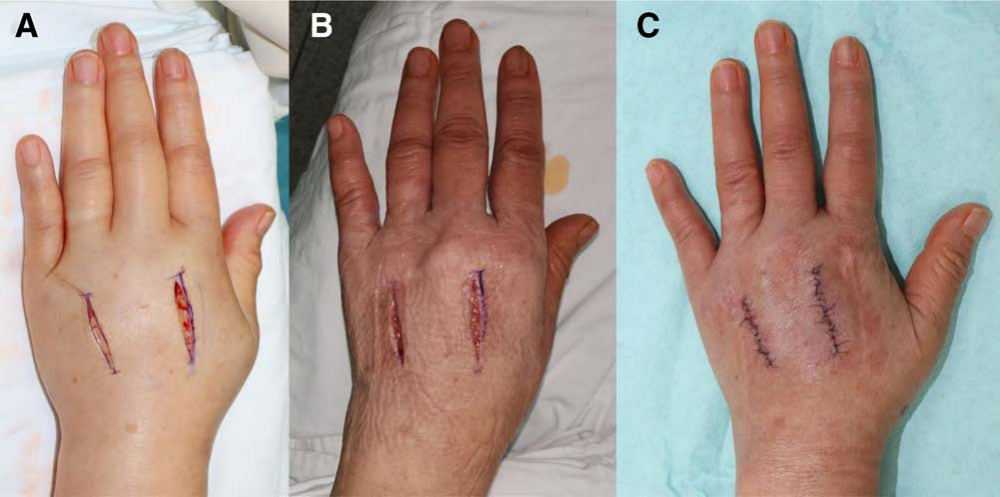
Surgical decompression and early postoperative recovery. (A) Prophylactic fasciotomy performed through 2 dorsal incisions over the second and fourth metacarpal bones; negative pressure wound therapy (NPWT) was applied immediately after decompression. (B) Postoperative day 3: swelling significantly subsided, with clinical improvement in pain and sensation. (C) Primary wound closure performed on the same day without the need for skin grafting.

On hospital day 2 (post-injury day 1), a prophylactic fasciotomy was performed via 2 longitudinal dorsal incisions over the second and fourth metacarpal bones (Fig. 3A). All 4 dorsal interosseous compartments were decompressed. Intraoperatively, marked subcutaneous edema and venous congestion were noted. However, no muscle necrosis or irreversible ischemic damage was observed. NPWT was applied immediately postoperatively using the CuraVAC system (CG Bio, Korea) set to continuous, 100 mm Hg suction. By postoperative day 3, the dorsal hand swelling had markedly improved (Fig. 3B), and the patient reported near-complete resolution of pain and paresthesia. On the same day, the fasciotomy wounds were closed without the need for skin grafting (Fig. 3C). No complications were observed during wound closure.

**Figure 4.**
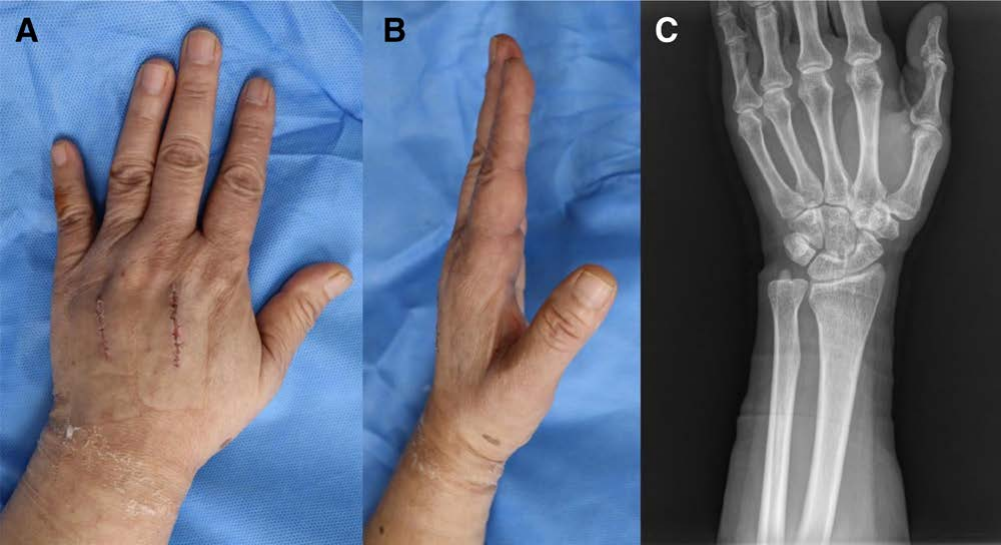
Final outcome on postoperative day 18. (A) Dorsal view of the left hand after suture removal showing well-healed incisions with minimal scarring. (B) Lateral view demonstrating full soft tissue recovery and restoration of contour. (C) Follow-up radiograph showing a complete resolution of soft tissue edema and absence of structural complications.

On postoperative day 18, the patient returned for an outpatient evaluation following suture removal. The fasciotomy wounds had healed completely with minimal scarring (Fig. 4A and B), and the patient reported no residual pain, numbness, or functional limitation. A follow-up radiograph confirmed complete soft tissue resolution with no delayed complications such as calcification, joint stiffness, or bone injury (Fig. 4C).

**Figure 5.**
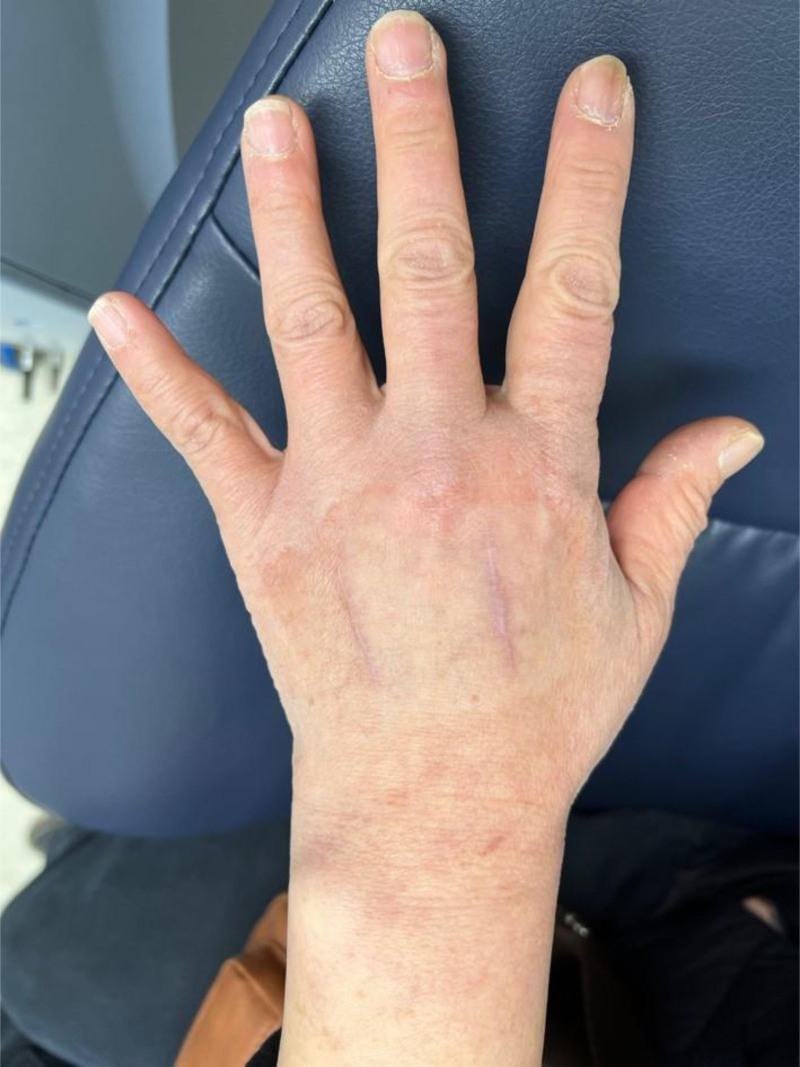
Clinical photograph at postoperative day 25 showing near-complete scar remodeling after suture removal. No hypertrophic scarring, pigmentation, or functional limitation is observed.

By postoperative day 25, the scars had become barely perceptible, and the patient remained fully asymptomatic with no restrictions in daily hand use. The esthetic and functional outcomes were excellent in the final stage of recovery (Fig. 5).

## 3. Discussion

Although contrast medium extravasation is often regarded as a self-limiting complication of intravenous imaging, certain anatomical regions such as the dorsum of the hand present unique risks because of their confined fascial compartments and limited capacity for expansion.^[1]^ When a large volume of contrast infiltrates such regions, tissue perfusion may be critically compromised, even in the absence of overt skin changes. In these high-risk settings, delayed or missed recognition of early ischemia may lead to irreversible soft tissue damage.^[3]^ Thus, adjunctive diagnostic tools are needed to supplement clinical judgment and identify patients who would benefit from early surgical intervention.^[4]^

TcPO is widely used as a noninvasive tool to assess tissue perfusion in chronic ischemic conditions, particularly in diabetic foot ulcers and peripheral arterial disease. Values below 20 mm Hg have been consistently associated with delayed wound healing and critical ischemia.^[7]^ While these data are primarily derived from chronic ischemic wounds, no standardized threshold has yet been established for acute extravasation injuries. In the present case, we therefore applied a threshold of 20 mm Hg as a precautionary marker of severe ischemia, with the aim of preventing irreversible tissue damage. TcPO₂ has also shown comparable results to FLIR thermography in assessing local perfusion, supporting its value as a complementary noninvasive modality in clinical settings.^[8]^ These findings collectively support the broader applicability of TcPO₂ across a range of ischemic conditions.

In the present case, TcPO₂ monitoring served as the primary objective tool to evaluate local tissue perfusion. Critically low values (1–5 mm Hg) were recorded despite the absence of classic ischemic signs such as skin discoloration, blistering, or delayed capillary refill. This discrepancy between clinical appearance and perfusion status illustrates the diagnostic limitations of subjective examinations alone and highlights the value of TcPO₂ in detecting occult hypoxia before irreversible tissue damage occurs.^[6]^

To further support the clinical decision-making process in determining a differential diagnosis, we employed adjunctive thermographic imaging using a handheld infrared camera. The surface temperature of the affected area was ~33°C, comparable to the surrounding skin and within the normal physiologic range, thereby excluding focal hyperthermia suggestive of inflammation or infection. Infrared thermography has been validated in clinical practice for identifying superficial inflammation and early soft tissue infection.^[9]^ This dual-modality approach, combining TcPO₂ and thermography, enabled a more nuanced assessment of tissue viability and demonstrated markedly decreased TcPO₂ (<20 mm Hg) with compromised perfusion. In the context of the patient’s rapid clinical deterioration, these findings were obtained in the absence of overt external signs and justified our decision to proceed with immediate prophylactic fasciotomy to prevent permanent tissue injury.

Although the fasciotomy was performed ~24 hours after the initial injury, beyond the often-cited 6-hour “golden window” for compartment syndrome,^[10]^ objective hypoxia guided our decision rather than elapsed time alone. This case underscores that TcPO₂ may be particularly valuable in delayed or ambiguous presentations, in which traditional clinical signs are insufficient to guide management. Even in the absence of irreversible ischemic damage, TcPO₂ provides a real-time quantification of tissue viability and may prevent both overtreatment and missed intervention opportunities.^[11]^

Additionally, the application of NPWT after decompression served to control edema, stabilize the wound environment, and accelerate granulation tissue formation. Although NPWT is widely used for managing fasciotomy wounds in trauma or vascular contexts,^[12]^ its use in contrast extravasation injuries has rarely been reported. In the present case, a postoperative 3-day course of NPWT facilitated safe primary closure without the need for skin grafting, suggesting that this modality could be a valuable treatment adjunct to improve postoperative recovery in similar cases.^[13]^

By postoperative day 25, the patient showed favorable recovery, with scarcely visible scars and full return to daily hand use without discomfort. However, the patient did not return for further visits, which limits the ability to draw conclusions regarding the long-term efficacy and durability of this treatment approach. While this represents an inherent limitation of the present report, the encouraging recovery observed supports the potential value of perfusion-guided early intervention. Future studies with extended follow-up and larger cohorts will be necessary to better define long-term outcomes in similar cases. In addition, the lack of standardized recovery metrics in this context may limit broader applicability, highlighting the importance of future prospective validation.

This case highlights 2 important clinical insights: first, TcPO₂, particularly when combined with thermal imaging, may assist in early risk stratification of contrast extravasation injuries in high-risk anatomical zones; second, perfusion-guided early fasciotomy can potentially prevent the development of full-blown compartment syndrome. TcPO₂ and FLIR thermography may serve as useful diagnostic adjuncts to support early decision-making for fasciotomy in equivocal cases in which the traditional signs are absent. Although further prospective studies are warranted to refine the threshold values and clinical algorithms, this report provides a foundation for perfusion-based decision-making in the management of high-volume contrast extravasation injuries.

## 4. Conclusion

In summary, this case demonstrates the potential role of TcPO₂ monitoring in guiding early surgical decisions and intervention for contrast medium extravasation injuries. In the absence of clear clinical signs, TcPO₂ and FLIR thermography may improve timely decision-making for fasciotomy. To further validate the clinical utility of this approach, we plan to accumulate additional cases and share the subsequent findings in future reports.

## Author contributions

**Conceptualization:** Chan Yeong Lee.

**Data curation:** Hwan Jun Choi.

**Formal analysis:** Hwan Jun Choi.

**Methodology:** Woo Jin Song.

**Project administration:** Hwan Jun Choi.

**Supervision:** Hwan Jun Choi.

**Visualization:** Hwan Jun Choi.

**Writing – original draft:** Hyun Beom Choi.

**Writing – review & editing:** Ha Jong Nam, Hwan Jun Choi.
